# Radiographic Characteristics of Feline Nasopharyngeal Stenosis

**DOI:** 10.1111/vru.70072

**Published:** 2025-08-14

**Authors:** Ayano Masuyama, Masahiro Suematsu, Caroline Fulkerson, Tetsuya Taniguchi, Masaya Nakamori, Kanami Nakao, Masahiro Murakami

**Affiliations:** ^1^ Department of Veterinary Clinical Sciences College of Veterinary Medicine, Purdue University West Lafayette Indiana USA; ^2^ AMC Suematsu Animal Hospital Oita Japan; ^3^ Hyogo Pet Medical Center Kobe Japan; ^4^ Kyoto Animal Medical Center Kyoto Japan

**Keywords:** respiratory diseases, soft palate, stertor, upper airway obstruction

## Abstract

Feline nasopharyngeal stenosis (NPS), characterized by narrowing of the nasopharyngeal passage, results in chronic stertor and increased inspiratory effort. While rhinoscopy is the definitive diagnostic modality for NPS, the utility of nasopharyngeal radiography in diagnosis remains under‐documented. The purpose of this study is to evaluate the diagnostic accuracy and describe the characteristic radiographic findings in cats with NPS by comparing radiographic findings in cats with stertor but without stenosis. This is a multicenter, retrospective, cross‐sectional study. Fifty‐two cats with stertor who underwent both rhinoscopy and lateral nasopharyngeal radiography were included. They were divided into two groups: those with NPS (*n* = 21) and those with stertor without stenosis (ND group, *n* = 31). Radiographs were reviewed by two board‐certified radiologists to determine the presence, location, and morphology (broad or membranous) of NPS, as well as the morphology of the soft palate and the presence of oropharyngeal gas. Radiographic evaluation demonstrated a high diagnostic accuracy for NPS with a sensitivity of 100%, specificity of 83.9%, and overall accuracy of 90.4%. The radiographic morphology of the NPS was broad in 18 cats and membranous in 3 cats. A “bent” soft palate was observed only in the NPS group (19%, *n* = 4). The presence of oropharyngeal gas was similar in both groups (57.1 vs. 58.1%). However, because radiographic diagnoses were made by consensus between two radiologists, the reported accuracy may be overestimated. Given its high sensitivity, nasopharyngeal radiography serves as an effective initial screening tool for NPS, likely streamlining the diagnostic pathway in cats with stertor.

## 1 Introduction

Nasopharyngeal stenosis (NPS) is a disease characterized by narrowing of the nasopharyngeal passage, resulting in upper airway obstruction with chronic stertor and increased inspiratory effort [1, 2, 3, 4]. Although it occasionally occurs as a congenital anomaly in dogs and cats [5, 6, 7, 8], it is more commonly a sequela of inflammatory conditions. Such conditions include rhinitis [7, 9, 10], infection [7, 11], trauma [12], and postsurgical procedures [7, 10], which can result in the formation of scar tissue within the nasopharynx.

Confirmatory diagnosis of NPS is usually made by retroflex rhinoscopy of the nasopharynx using a flexible endoscope [8, 12, 13, 14, 15, 16] to exclude neoplastic lesions or foreign bodies in the nasopharynx and to confirm the presence of a narrowed or membranous structure causing stenosis. CT is also used to evaluate the nasopharynx, diagnose NPS, and assess the severity of the disease [8, 17, 18, 19]. Lateral radiographs of the head and pharynx are often obtained as a preliminary study before proceeding to more advanced imaging modalities such as endoscopy or CT [13, 14].

While several case reports have described radiographic changes associated with feline NPS [12, 14, 20, 21, 22], a comprehensive evaluation of radiographic features has not been reported. Radiographic assessment of feline NPS in primary veterinary practice, especially in cats exhibiting clinical signs of upper airway obstruction such as stertor, could streamline the process of referring such cases for more advanced diagnostic procedures for confirmatory diagnosis and more appropriate treatment.

The purpose of this study is to describe characteristic radiographic findings in cats with NPS and to compare them with cats with stertor without NPS. We hypothesized that (1) radiographic diagnosis of NPS in cats would be highly sensitive but less specific, (2) on the radiograph, cats with NPS would commonly show a broad narrowing of the nasopharynx, dorsal deviation of the soft palate with “bent” morphology, and continuous gas in the oropharynx on lateral radiographs compared with cats without NPS.

## 2 Materials and Methods

### 2.1 Case Selection

This study was designed as a multicenter, retrospective, cross‐sectional, observational study. The retrospective search was conducted from November 2019 to April 2023, and medical records from three referral veterinary hospitals in Japan were reviewed for inclusion. As this study was retrospective and utilized pre‐existing medical records and imaging data, approval from the Institutional Animal Care and Use Committee (IACUC) was not required.

The retrospective case series consisted of cats that presented with stertor and underwent diagnostic imaging, including lateral radiography of the head and pharynx during the inspiratory phase, followed by rhinoscopic evaluation of the nasopharynx.

We divided the cases into two groups based on rhinoscopic findings: the NPS group, consisting of cats with a confirmed diagnosis of NPS, and the nasal disease (ND) group, consisting of cats with normal rhinoscopic findings of the nasopharynx without evidence of stenosis. Exclusion criteria included cases with nasopharyngeal masses or structures, including nasopharyngeal polyps.

Clinical details such as breed, age, sex, and body weight were thoroughly documented by the veterinary staff at the participating hospitals. Consent for the use of medical records in this research was obtained from the respective hospital owner/director and cat owners.

Cases were excluded if lateral radiographs prior to rhinoscopy were of suboptimal quality, misaligned, or absent. Eligibility of cases for inclusion or exclusion was determined by an American College of Veterinary Radiology (ACVR) board‐certified radiologist (M. M.) and a veterinarian with 15 years of experience specializing in respiratory medicine (M. S.).

### 2.2 Radiographic Interpretation

**FIGURE 1:**
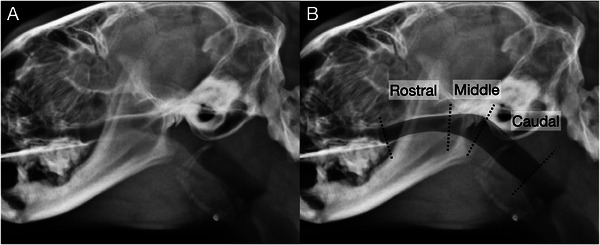
Lateral radiograph of the nasopharynx in a cat without evidence of NPS. (A) Unannotated radiograph. (B) Annotated radiograph with the nasopharynx shaded in gray and dotted lines demarcating the rostral, middle, and caudal portions of the nasopharynx. The middle portion of the nasopharynx is defined by the location of the temporomandibular joint.

**FIGURE 2:**
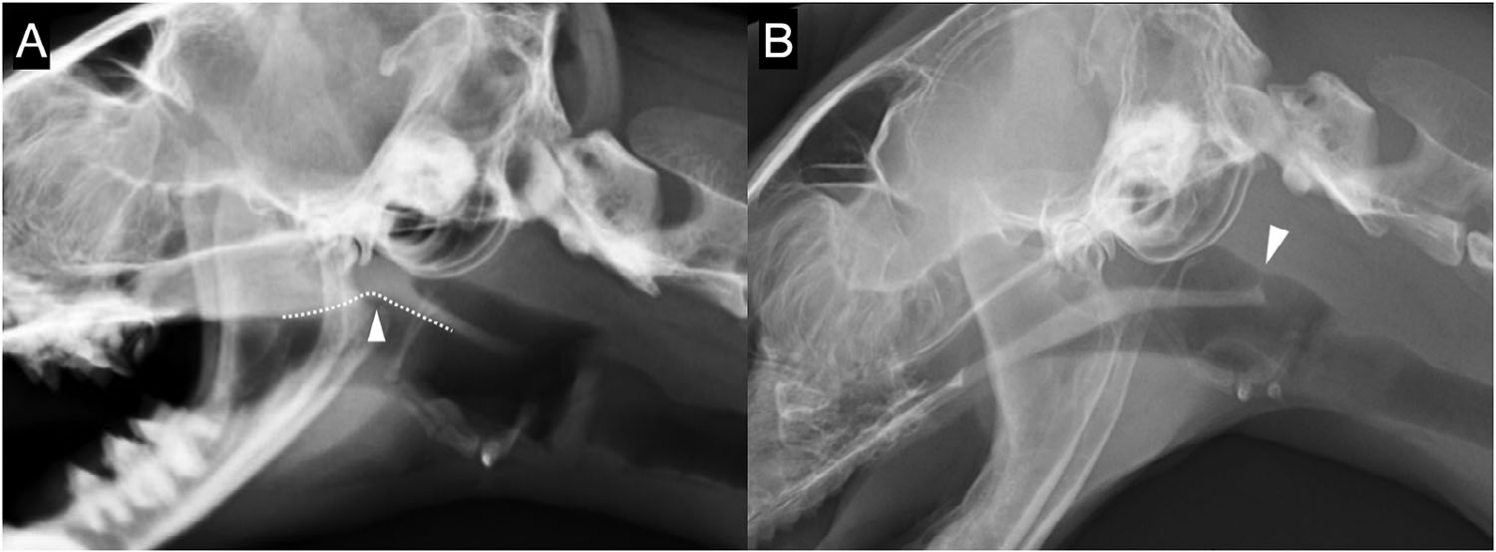
Lateral radiographs of the nasopharynx in two cats with NPS. (A) Broad stenosis involving the rostral and middle portions of the nasopharynx (categorized as middle due to inclusion at the level of the temporomandibular joint) with a “bent” soft palate morphology (dotted line and white arrowhead). (B) Membranous stenosis (white arrowhead) in the caudal nasopharynx with normal soft palate morphology.

**FIGURE 3:**
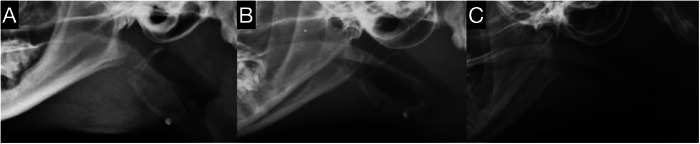
Lateral radiographs of the nasopharynx in cats without NPS. (A) Absence of gas within the oropharynx. (B) Partial presence of gas within the oropharynx. (C) Continuous presence of gas within the oropharynx.

The radiographs were randomized by one of the authors (A. M.) and reviewed by two ACVR board‐certified radiologists (C. F. and M. M.), who were blinded to the diagnosis. Consensus on the findings was reached using DICOM viewer software (Osirix MD, Bernex, Switzerland, v.12.0.1). Lateral radiographs of the head and pharynx in the inspiratory phase were evaluated in all groups. Subjective analysis aimed to identify the presence and location of NPS (categorized as rostral, middle, or caudal; Figure 1), describe its morphology (broad or membranous; Figure 2) and soft palate morphology (Figure 2A), and determine the presence of gas in the oropharynx (Figure 3). NPS was defined as a soft tissue opaque structure dividing the nasopharyngeal gas opacity in a rostrocaudal direction. The location of the NPS was classified as middle if the soft tissue opacity caused the stenosis at the level of the temporomandibular joint, and as rostral or caudal if it was rostral or caudal to the temporomandibular joint, respectively (Figure 1). A normal morphology of the soft palate was identified by its smooth margin with slight ventral curvature. Soft palates with a sharp ventral curvature at the oropharyngeal margin were classified as “bent”, which is a subjective assessment (Figure 2A). The presence of gas in the oropharynx was documented as “continuous” if it completely separated the soft palate from the tongue or mandibular soft tissue, and as “partial” if this separation was incomplete (Figure 3).

### 2.3 Statistics

Descriptive statistics, including mean and standard deviation (SD), were calculated for continuous variables, such as age and body weight. Categorical variables, such as breed and sex, were expressed as the number and percentage.

We calculated sensitivity, specificity, positive predictive value, negative predictive value, and accuracy for radiographic diagnosis of NPS. In addition, we determined the proportions (%) of stenosis location and type, soft palate morphology, and presence of oropharyngeal gas within each group.

## 3 Result

### 3.1 Case Information

A total of 52 cats were included and subjected to the radiographic interpretation, with 21 cats classified into the NPS group and 31 cats into the ND group. The mean age of the cats in the NPS group was 9.0 (SD: 4.3) years, and in the ND group was 6.9 (SD: 5.1) years. The body weight ranged from 2.6 to 5.8 kg (mean: 4.0 kg, SD: 1.0) for the NPS group and 1.5 to 6.2 kg (mean: 4.0 kg, SD: 1.1) for the ND group. The distribution of breeds was similar across both groups, with 85% being domestic shorthairs.

### 3.2 Radiographic Evaluation and Diagnostic Accuracy

**FIGURE 4:**
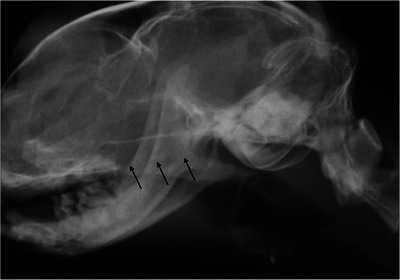
Lateral radiograph of the nasopharynx in a cat without NPS. However, a false‐positive diagnosis of NPS was made radiographically. Black arrows indicate the region of the pterygoid processes of the basisphenoid bone.

The diagnostic accuracy of lateral radiographic interpretation for NPS yielded a sensitivity of 100%, specificity of 83.9%, positive predictive value of 80.8%, negative predictive value of 100%, and an overall accuracy of 90.4%. True positive radiographic diagnoses were observed in all 21 cats in the NPS group, while the ND group accounted for 26 true negatives and 5 false positives (Figure 4). There were no false negative results.

### 3.3 Localization and Morphology of NPS

Of the 26 cats in which radiographic NPS was suspected, 21 were true positives from the NPS group. Radiographic stenosis location in these true positive cats was identified as rostral in 9 cats (43%), middle in 6 cats (29%), and caudal in 6 cats (29%). The radiographic morphologic evaluation of the stenosis was broad in 18 cats and membranous in 3 cats (Figure 2). False positive diagnoses occurred in 5 cats, 4 with rostral (Figure 4) and 1 with middle NPS, all with broad stenosis (Figure 4).

### 3.4 Soft Palate Morphology

The “bent” soft palate appearance was noted in four cases (Figure 2A), representing 19% of the NPS group, and was only observed in the NPS group.

### 3.5 Oropharyngeal Gas Presence

The presence of oropharyngeal gas was identified in 57.1% of the NPS group, with partial gas in 7 cats (33.3%; Figure 3B) and continuous gas in 5 cats (23.8%; Figure 3C). In the ND group, oropharyngeal gas was similarly prevalent, with 58.1% displaying oropharyngeal gas, partial gas in 9 cats (29.0%), and continuous gas in 9 cats (29.0%).

## 4 Discussion

The present study demonstrated that lateral nasopharyngeal radiographs exhibit a high diagnostic accuracy for NPS in cats presenting with stertor, as evidenced by the sensitivity and negative predictive value of 100%. In accordance with our first hypothesis, a few cases in the ND group were incorrectly diagnosed with NPS, resulting in a relatively low specificity (83.9%) and positive predictive value (80.8%). The occurrence of false‐positive results within the ND group reinforces the need for confirmatory diagnostic techniques such as rhinoscopy. Our results suggest that radiography is an effective initial diagnostic tool for feline NPS with characteristic radiographic findings in cats with stertor.

Radiographic evaluation of the nasopharynx has limitations due to the superimposition and silhouetting of multiple soft tissues over mineral opaque structures [23, 24]. Especially when there isn't enough gas within the nasopharynx, it is hard to differentiate true NPS from a collapsed nasopharynx, leading to increased false‐positive results.

Another potential cause of false‐positive diagnoses is the superimposition of the pterygoid processes of the basisphenoid bone (Figure 4). These paired ventral projections of the basisphenoid are thin, sagittal plates measuring approximately 1 cm in width and length, and spaced slightly more than 1 cm apart [25]. Due to their morphology and location, the pterygoid processes can be radiographically misinterpreted as a soft tissue‐opaque structure within the nasopharynx, potentially mimicking NPS.

The nasopharynx is a part of the upper airway tract extending from the choanae to the pharyngeal isthmus. The rostral part of the nasopharynx is delineated by the hard palate ventrally, the vomer dorsally, and the palatine bones bilaterally. The middle and caudal portions of the nasopharynx are bounded dorsally by the base of the skull and the muscles attaching to it, and ventrally by the soft palate [25, 26, 27]. NPS is a pathologic narrowing of the nasopharynx leading to upper airway obstruction [1, 2, 3, 4]. It is a rare condition in domestic animals, accounting for 1.5% of the pharyngeal diseases in dogs [26] and 6% of the nasal diseases in cats [13]. Previous studies showed that the common location of the NPS in cats is the caudal nasopharynx, with 66–83% of the lesions being located in the caudal part of the nasopharynx in cats with NPS or imperforate nasopharynx [7, 14, 18]. On the other hand, another study showed that four out of six cats with NPS had stenotic lesions more cranially, arising from the caudal aspect of the hard palate [17]. In the present study, the majority of the stenosis locations were radiographically identified as rostral, relative to the temporomandibular joint, and only 29% of the lesions were identified as caudal. However, as the detailed rhinoscopy results or CT images were not available in our study, the actual locations or extension of the stenotic lesions were unknown.

In animals with NPS, a thin membrane or fibrous granulation tissue obstructs the nasopharynx, causing signs indicating upper airway obstruction [28]. These lesions may be observed as a membranous structure or a broad narrowing of the nasopharynx in the radiographs [12, 14, 22]. Although there have been sporadic reports mentioning the morphology of the NPS, no comprehensive study has been attempted so far. Some studies sporadically reported a strand‐like, thin, soft tissue opaque stenotic membrane within the nasopharynx in radiographs of cats with NPS [12, 21]. Recent studies described CT findings of the stenotic lesions either as a soft tissue membrane and/or partial or complete adhesion of the soft palate and the nasopharynx [4, 18]. This study reported that the average length of the stenotic lesions was 3.02 mm [18], which might be identified as a membranous structure in radiographs. On the other hand, another CT study reported that the length of stenotic lesions varied from 4.5 to 18 mm [17], which might be identified as a membrane or a broad narrowing in radiographs. In the present study, a broad narrowing of the nasopharynx was frequently observed in cats with NPS, supporting our second hypothesis. This finding may be explained by the fact that most cats in our study population likely had extensive adhesion between the soft palate and nasopharynx, or that soft tissue surrounding the nasopharynx silhouetted the area of stenotic lesions, giving the appearance of broader stenosis, even in cases of membranous stenosis. Due to the retrospective nature of this study, detailed rhinoscopy results were unavailable, leaving the exact extent of stenotic areas in our population unknown.

Other radiographic findings related to NPS in cats reported included dorsal deviation of the soft palate [14, 20, 21]. The first study that reported the radiographic appearance of the nasopharynx of a cat with NPS described the dorsal deviation of the soft palate [20]. The following study reported that 10 out of 15 cats with NPS showed dorsal deviation or the deformation of the soft palate [14]. In our study, a dorsal deviation of the soft palate with a “bent” morphology was specifically observed in cats with NPS, with a prevalence of 19%. However, this assessment was subjective, and the authors noted that a pronounced curvature of the soft palate observed in cats with a slightly ventrally flexed head position made it challenging to distinguish true “bent” morphology from severe curvature. This limitation stemmed from the retrospective nature of the study. Future studies utilizing lateral radiographs obtained with standardized, straight head and neck positioning in all cases may provide a more accurate determination of the prevalence of this “bent” morphology.

Cats are known to be obligate nasal breathers, relying on their nasal passages for breathing under normal conditions. When significant upper airway obstruction occurs, they may exhibit open‐mouth breathing, a sign of severe respiratory distress [29]. For this reason, radiographic oropharyngeal distention is sometimes considered abnormal in cats to suggest respiratory distress. However, the presence of oropharyngeal gas as a diagnostic marker in feline respiratory disease remains poorly understood. In the present study, more than half of the cats, both with and without NPS, exhibited oropharyngeal gas. This feature was not specific to NPS but may be associated with upper airway obstruction, as stertor was observed in both groups. To further assess the clinical importance of oropharyngeal gas in cats relative to respiratory distress, additional studies are needed to compare its occurrence in cats with and without stertor or other respiratory conditions.

Our study has several limitations, mainly due to its retrospective design and small number of cases. A main limitation was the lack of detailed rhinoscopic evaluation and the absence of CT imaging, which prevented precise determination of the location and morphology of the stenotic lesions. In addition, head and neck positioning was not standardized, potentially affecting the radiographic appearance of gas distribution and soft palate morphology. Additionally, radiographic diagnoses were determined by consensus between two board‐certified radiologists, preventing assessment of inter‐ or intraobserver variability. Consequently, our reported diagnostic accuracy may be overestimated, as we did not quantify how consistently different observers (or the same observers at different times) would interpret the same images. Because our current radiologists already know the definitive diagnosis, repeating the review with these same observers to measure reliability would not be feasible. Future studies incorporating multiple observers with varying levels of training, as well as comprehensive evaluations of NPS, including advanced imaging techniques and standardized radiographic positioning, are needed to enhance our understanding of this condition and clarify the effects of observer variation on diagnostic accuracy.

In conclusion, lateral nasopharyngeal radiographs demonstrated high diagnostic accuracy with high sensitivity and high negative predictive value for NPS in cats presenting with stertor. Radiographic diagnosis was strongly associated with true positive findings in the NPS group, more commonly with broad‐type stenosis and a few cases of membrane‐type stenosis. Although prevalence is relatively low, the membranous structure crossing the nasopharynx and the “bent” morphology of the soft palate were specifically observed in cats in the NSP group, suggesting that these radiographic findings can be characteristics of the feline NPS. Due to the false‐positive results within the ND group, advanced diagnostic modalities such as rhinoscopy are needed for confirmation. Thus, radiography is a good initial diagnostic modality for feline NPS with characteristic radiographic findings in cats with stertor.

## Author Contributions

Category 1

- Conception and design: Murakami, Taniguchi, Nakamori, Suematsu
- Acquisition of data: Masuyama, Murakami, Fulkerson, Taniguchi, Nakamori, Nakao, Suematsu
- Analysis and interpretation of data: Masuyama, Murakami, Fulkerson, Suematsu

Category 2

- Drafting the article: Masuyama, Murakami, Suematsu
- Revising article for intellectual content: Masuyama, Murakami, Fulkerson, Taniguchi, Nakamori, Nakao, Suematsu

Category 3

- Final approval of the completed article: Masuyama, Murakami, Fulkerson, Taniguchi, Nakamori, Nakao, Suematsu

Category 4

- Agreement to be accountable for all aspects of the work in ensuring that questions related to the accuracy or integrity of any part of the work are appropriately investigated and resolved: Masuyama, Murakami, Fulkerson, Taniguchi, Nakamori, Nakao, Suematsu

## Conflicts of Interest

The authors declare no conflicts of interest.

## Previous Presentation or Publication Disclosure

The abstract of this study was presented at the 2024 Annual Conference of the American College of Veterinary Radiology, held in Norfolk, Virginia, USA.

## EQUATOR Network Disclosure

An EQUATOR network checklist was not used.

## References

[1] A. C. Berent , “Diagnosis and Management of Nasopharyngeal Stenosis,” The Veterinary Clinics of North America. Small Animal Practice 46 (2016): 677–689. 27059368 10.1016/j.cvsm.2016.01.005

[2] N. F. Kuehn , “Chronic Rhinitis in Cats,” Clinical Techniques in Small Animal Practice 21 (2006): 69–75. 16711612 10.1053/j.ctsap.2005.12.013

[3] S. Z. Pollack , P. S. Chapman , and A. Klag , “Balloon Dilation for the Treatment of Nasopharyngeal Stenosis in Seven Cats,” Journal of Feline Medicine and Surgery Open Reports 3 (2017): 2055116917729987. 28955477 10.1177/2055116917729987 PMC5607926

[4] P. Sériot , S. Gibert , L. Poujol , F. Bernardin , L. Blond , and A. Dunié‐Mérigot , “Extended Palatoplasty as Surgical Treatment for Nasopharyngeal Stenosis in Six Cats,” Journal of Small Animal Practice 60 (2019): 559–564. 31259420 10.1111/jsap.13048

[5] J. Talavera Lopez , M. A. Josefa Fernandez Del Palacio , F. G. Cano , and A. B. Del Rio , “Nasopharyngeal Stenosis Secondary to Soft Palate Dysgenesis in a Cat,” Veterinary Journal 181 (2009): 200–204. 18417393 10.1016/j.tvjl.2008.02.026

[6] B. R. Coolman , S. M. Marretta , B. C. McKiernan , and J. F. Zachary , “Choanal Atresia and Secondary Nasopharyngeal Stenosis in a Dog,” Journal of the American Animal Hospital Association 34 (1998): 497–501. 9826286 10.5326/15473317-34-6-497

[7] S. Burdick , A. C. Berent , C. Weisse , et al., “Interventional Treatment of Benign Nasopharyngeal Stenosis and Imperforate Nasopharynx in Dogs and Cats: 46 Cases (2005‐2013),” Journal of the American Veterinary Medical Association 253 (2018): 1300–1308. 30398419 10.2460/javma.253.10.1300

[8] D. M. DeSandre‐Robinson , S. N. Madden , and J. T. Walker , “Nasopharyngeal Stenosis With Concurrent Hiatal Hernia and Megaesophagus in an 8‐year‐old Cat,” Journal of Feline Medicine and Surgery 13 (2011): 454–459. 21334235 10.1016/j.jfms.2011.01.007 PMC10832710

[9] A. C. Berent , J. Kinns , and C. Weisse , “Balloon Dilatation of Nasopharyngeal Stenosis in a Dog,” Journal of the American Veterinary Medical Association 229 (2006): 385–388. 16881830 10.2460/javma.229.3.385

[10] A. K. Cook , K. T. Mankin , A. B. Saunders , C. E. Waugh , L. C. Cuddy , and G. W. Ellison , “Palatal Erosion and Oronasal Fistulation Following Covered Nasopharyngeal Stent Placement in Two Dogs,” Irish Veterinary Journal 66 (2013): 8. 23635357 10.1186/2046-0481-66-8 PMC3645959

[11] R. W. Mitten , “Nasopharyngeal Stenosis in Four Cats,” Journal of Small Animal Practice 29 (1988): 341–345.

[12] N. Reed and D. Gunn‐Moore , “Nasopharyngeal Disease in Cats: 2. Specific Conditions and Their Management,” Journal of Feline Medicine and Surgery 14 (2012): 317–326. 22511474 10.1177/1098612X12444998 PMC11132258

[13] S. M. Henderson , K. Bradley , M. J. Day , et al., “Investigation of Nasal Disease in the Cat–a Retrospective Study of 77 Cases,” Journal of Feline Medicine and Surgery 6 (2004): 245–257. 15265480 10.1016/j.jfms.2003.08.005 PMC10822601

[14] D. De Lorenzi , D. Bertoncello , S. Comastri , and E. Bottero , “Treatment of Acquired Nasopharyngeal Stenosis Using a Removable Silicone Stent,” Journal of Feline Medicine and Surgery 17 (2015): 117–124. 24820997 10.1177/1098612X14533692 PMC10816422

[15] T. M. Glaus , K. Tomsa , and C. E. Reusch , “Balloon Dilation for the Treatment of Chronic Recurrent Nasopharyngeal Stenosis in a Cat,” Journal of Small Animal Practice 43 (2002): 88–90. 11873954 10.1111/j.1748-5827.2002.tb00036.x

[16] T. M. Glaus , B. Gerber , K. Tomsa , M. Keiser , and S. Unterer , “Reproducible and Long‐lasting Success of Balloon Dilation of Nasopharyngeal Stenosis in Cats,” The Veterinary Record 157 (2005): 257–259. 16127136 10.1136/vr.157.9.257

[17] A. C. Berent , C. Weisse , K. Todd , M. P. Rondeau , and A. M. Reiter , “Use of a Balloon‐Expandable Metallic Stent for Treatment of Nasopharyngeal Stenosis in Dogs and Cats: Six Cases (2005‐2007),” Journal of the American Veterinary Medical Association 233 (2008): 1432–1440. 18980496 10.2460/javma.233.9.1432

[18] L. Mestrinho and R. Fonseca , “Clinical and Computed Tomography Findings in Cats With Nasopharyngeal Stenosis,” Journal of Small Animal Practice 65 (2024): 667–674. 38733276 10.1111/jsap.13739

[19] H. S. Allen , J. Broussard , and K. Noone , “Nasopharyngeal Diseases in Cats: A Retrospective Study of 53 Cases (1991‐1998),” Journal of the American Animal Hospital Association 35 (1999): 457–461. 10580903 10.5326/15473317-35-6-457

[20] D. J. Griffon and S. Tasker , “Use of a Mucosal Advancement Flap for the Treatment of Nasopharyngeal Stenosis in a Cat,” Journal of Small Animal Practice 41 (2000): 71–73. 10701190 10.1111/j.1748-5827.2000.tb03166.x

[21] A. Boswood , C. R. Lamb , D. J. Brockman , P. Mantis , and A. L. Witt , “Balloon Dilatation of Nasopharyngeal Stenosis in a Cat,” Veterinary Radiology & Ultrasound 44 (2003): 53–55. 12620051 10.1111/j.1740-8261.2003.tb01449.x

[22] R. E. Novo and B. Kramek , “Surgical Repair of Nasopharyngeal Stenosis in a Cat Using a Stent,” Journal of the American Animal Hospital Association 35 (1999): 251–256. 10333266 10.5326/15473317-35-3-251

[23] G. Carozzi , A. Zotti , M. Alberti , and F. Rossi , “Computed Tomographic Features of Pharyngeal Neoplasia in 25 Dogs,” Veterinary Radiology & Ultrasound 56 (2015): 628–637. 26173553 10.1111/vru.12278

[24] D. E. Thrall and I. D. Robertson , Atlas of Normal Radiographic Anatomy and Anatomic Variants in the Dog and Cat (Elsevier Health Sciences, 2015).

[25] J. W. Hermanson and A. De Lahunta , Miller and Evans' anatomy of the Dog (Elsevier Health Sciences, 2018).

[26] F. Billen , M. J. Day , and C. Clercx , “Diagnosis of Pharyngeal Disorders in Dogs: A Retrospective Study of 67 Cases,” Journal of Small Animal Practice 47 (2006): 122–129. 16512843 10.1111/j.1748-5827.2006.00032.x

[27] D. E. Thrall and W. R. Widmer , Textbook of Veterinary Diagnostic Radiology (St. Louis, Missouri: Saunders, 2018).

[28] T. W. Maddox , “Skull—nasal Chambers and Frontal sinuses,” BSAVA Manual of Canine and Feline Musculoskeletal Disorders (BSAVA Library, 2018): 301–315.

[29] D. L. Clarke , “Upper Airway Disease,” Feline Emergency and Critical Care Medicine (2022): 109–118.

